# Alveolar air and oxidative metabolic demand during exercise in healthy adults: the role of single‐nucleotide polymorphisms of the *β*_2_ AR gene

**DOI:** 10.14814/phy2.13476

**Published:** 2017-10-23

**Authors:** Erik H. Van Iterson, Eric M. Snyder, Bruce D. Johnson

**Affiliations:** ^1^ Department of Cardiovascular Medicine Mayo Clinic College of Medicine Rochester Minnesota; ^2^ Department of Kinesiology University of Minnesota Minneapolis Minnesota

**Keywords:** Aerobic exercise, codon 16, exercise capacity, genetic polymorphism, *β*_2_‐adrenergic receptor

## Abstract

The predominating *β*‐adrenergic receptor subtype expressed on human alveolar tissue is the *β*_2_ AR. The homozygous arginine (Arg16Arg) single‐nucleotide polymorphism (SNP) at codon 16 of the *β*_2_ AR gene has been associated with abnormal *β*_2_ AR function accompanied by decreased resting alveolar‐capillary membrane gas‐transfer in certain healthy adults. Although not previously studied in the context of the *β*_2_ AR gene, pulmonary gas‐transfer is also influenced by alveolar volume (*V*_A_) and with it the availability of alveolar surface area, particularly during exercise. Small *V*_A_ implies less alveolar surface area available for O_2_ transport. We tested the following hypothesis in healthy adults during exercise: compared with Gly16Gly and Arg16Gly β2AR genotypes, Arg16Arg will demonstrate reduced *V*_A_ and ventilation (*V̇*_A_) relative to *V̇*_E_ and oxidative metabolic demand. Age‐ BMI‐ and gender‐matched groups of Arg16Arg (*N* = 16), Gly16Gly (*N* = 31), and Arg16Gly (*N* = 17) performed consecutive low (9‐min, 40%‐peak workload) and moderate (9‐min, 75%‐peak workload) intensity exercise. We derived *V*_A_ and *V̇*_A_ using “ideal” alveolar equations via arterialized gases combined with breath‐by‐breath ventilation and gas‐exchange measurements; whereas steady‐state *V̇*O_2_ was used in metabolic equations to derive exercise economy (EC = workload÷*V̇*O_2_). Variables at rest did not differ across *β*_2_ AR genotype. Strongest *β*_2_ AR genotype effects occurred during moderate exercise. Accordingly, while *V̇*_E_ did not differ across genotype (*P* > 0.05), decreased in Arg16Arg versus Arg16Gly and Gly16Gly were *V̇*O_2_ (1110 ± 263, 1269 ± 221, 1300 ± 319 mL/(min·m^2^), respectively, both *P* < 0.05), *V̇*_A_ (59 ± 21, 70 ± 16, 70 ± 21 L/min, respectively, both *P *<* *0.05), and *V*_A_ (1.43 ± 0.37, 1.95 ± 0.61, 1.93 ± 0.65 L, respectively, both *P *<* *0.05). Also reduced was EC in Arg16Arg versus Arg16Gly (*P *<* *0.05) and Gly16Gly (*P *>* *0.05) (1.81 ± 0.23, 1.99 ± 0.30, and 1.94 ± 0.26 kcal/(L·m^2^), respectively). Compared with Gly16Gly and Arg16Gly genotypes, these data suggest the Arg16Arg *β*_2_ AR genotype plays a role in the loss of oxidative metabolic efficiency coupled with an inadaptive *V*_A_ and, hence, smaller alveolar surface area available for O_2_ transport during submaximal exercise in healthy adults.

## Introduction

The *β*_2_‐adrenergic receptor (*β*_2_AR) is a G‐coupled protein expressed on nearly all cell types in the lung (Carstairs et al. 1985; Spina et al. 1989; Green et al. 1994). With receptor distribution and density increasing with each successive airway generation (Carstairs et al. 1985; Spina et al. 1989), *β*_2_ARs play a critical role in helping to maintain total alveolar surface area needed for gas exchange (Sakuma et al. 1994; Kerem et al. 1999; McGraw et al. 2001; Sartori et al. 2002; Mutlu et al. 2004). This is consistent with reports suggesting that while >90% of all *β*AR expression in the lung is associated with approximately 300–500 million alveoli, the predominating subtype in this location is the *β*_2_AR (Carstairs et al. 1985; Spina et al. 1989; Ochs et al. 2004).

However, as a consequence of Starling forces (Starling 1896), decreases in alveolar air volume followed by loss of alveolar surface area needed for gas exchange (e.g., convective and diffusive O_2_ transport) can occur when fluid accumulates in alveoli. These coupled events may be provoked by exercise and/or stays in extreme environmental conditions (Kerem et al. 1999; Crandall and Matthay 2001; McGraw et al. 2001; Sartori et al. 2002; Snyder et al. 2006d, 2007). In these settings, when hydrostatic pressure of pulmonary capillaries is higher than that of the interstitial space accompanied by interstitial fluid accumulation that exceeds the rate of fluid removal, an influx of fluid into alveoli may occur (Starling 1896; Lauweryns and Baert 1977; Wallin and Leksell 1994; Kerem et al. 1999; Snyder et al. 2006c, 2007). Nevertheless, activation of the *β*_2_AR second messenger pathway including downstream effects on epithelial sodium channels plays an important role in intra‐alveolar fluid clearance and maintenance of total alveolar surface area needed for gas exchange (Dumasius et al. 2001; McGraw et al. 2001; Factor et al. 2002; Sartori et al. 2002; Mutlu et al. 2004; Snyder et al. 2007).

In otherwise healthy adults when abnormal lung fluid clearance occurs, this has been attributed to impaired *β*_2_AR function linked to unique single‐nucleotide polymorphisms (SNPs) at codon 16 of the *β*_2_AR gene (ADRB2) (Snyder et al. 2007). Snyder et al. (2007) reported that compared with the homozygous glycine (Gly16Gly) *β*_2_AR genotype, healthy adults homozygous for arginine (Arg16Arg) demonstrated reduced alveolar‐capillary membrane conductance (D_M_) coinciding with decreased lung fluid clearance following rapid intravenous infusions of saline at rest. Whether observations at rest (Snyder et al. 2007) involving pulmonary limitations to gas exchange (e.g., O_2_ transfer) and SNPs of the ADRB2 translates to coupling between *β*_2_AR genotype with alveolar respiratory responses and substrate oxidative capacity during exercise remains unclear.

Provided the intrinsic sympathomimetic effect of exercise leads to activated *β*_2_ARs, it could be expected that physiological changes in key components of gas exchange, O_2_ transport, and oxidative capacity are not limited to independent effects associated with increased cardiac output (*Q̇*), vasodilation, and so on (Kjaer et al. 1985; Liggett et al. 1988; Large et al. 1997; Snyder et al. 2006a; Wolfarth et al. 2007). Airway factors such as alveolar ventilation (*V̇*_A_) and alveolar volume (*V*_A_) with respect to global lung responses of minute ventilation (*V̇*_E_) and tidal volume (*V*_T_) also play important roles in gas exchange and O_2_ transport (Farhi and Rahn 1955; Hey et al. 1966; Dempsey et al. 1984; Aaron et al. 1992). Thus, it is under these broad assumptions where ours and others' isolated genomics studies involving SNPs of the ADRB2 (Dishy et al. 2001; Garovic et al. 2003; Snyder et al. 2006a, 2007) can be taken to test the following hypothesis in this study: compared with healthy adults demonstrating the Gly16Gly or Arg16Gly SNP for the ADRB2, there will be a reduced *V*_A_ driving an inadequate *V̇*_A_ response relative to both *V̇*_E_ and metabolic demand (i.e., gross and net substrate oxidation) during submaximal exercise in individuals expressing the Arg16Arg *β*_2_AR genotype. This hypothesis generating study tested in healthy adults involving possible genotype↔phenotype interactions linking SNPs at codon 16 of the ADRB2 to alveolar mechanisms of O_2_ transport and oxidative capacity has potential clinical translational implications for patients with advanced cardiopulmonary diseases for whom *β*_2_ARs are targets for pharmacotherapies aimed to improve aerobic capacity (Nelson 1995; Wagoner et al. 2000; Snyder et al. 2006d).

## Methods

### Participants

Sixty‐four Caucasian adults were recruited to participate in this study. All individuals provided written informed consent prior to study participation. All aspects of this study were reviewed and approved by the Mayo Clinic Institutional Review Board and conformed to the Declaration of Helsinki.

Careful review of medical records demonstrated no participant in this study was diagnosed with a cardiovascular, cardiopulmonary, or neuromuscular disease that would confound study interpretations. Participants were also nonsmokers, not pregnant, not on prescribed medications, and not dependent on alcohol or narcotics. Participants in this study were genotyped and stratified into groups according to SNPs at codon 16 of the ADRB2. Although we have previously studied this sample to test the influence of SNPs at codon 16 of the ADRB2 on cardiovascular responses to exercise (Snyder et al. 2006a), aims of this study constitute testing an original hypothesis, presentation of original data, and a logical next step in this research line. We studied 16, 31, and 17 healthy adults who were homozygous for Arg (Arg16Arg), homozygous for Gly (Gly16Gly), or heterozygous for Arg and Gly (Arg16Gly), respectively, at codon 16 of the ADRB2.

### Protocol overview

Participants arrived at the General Clinical Research Center (GCRC) for a baseline screening visit where a pregnancy test was administered to women, blood testing for hemoglobin (Hb) and hematocrit (Hct) levels was given to rule out anemia, and resting flow volume loop spirometry was performed to assess airway function according to the guidelines of the American Thoracic Society (Miller et al. 2005). Participants also performed an incremental cardiopulmonary exercise test (CPET) to assess peak exercise workload, which was confirmed during a mirrored second CPET performed on study visit 2. Test‐retest reliability of our CPET from study day 1 to 2 was strong [Intraclass correlation coefficient (Weir 2005; Van Iterson et al. 2017a) across the sample for peak workload between study days 1 and 2 was 0.98 with lower and upper 95% confidence limits (CL): 0.95, 0.99]. Peak workload was used to determine submaximal exercise workloads to be performed for the final visit (study day 3).

Because it is suggested variance in dietary sodium levels can confound the interpretation of *β*_2_AR function (Kotanko et al. 1992), study visit 3 occurred while maintaining a salt‐neutral diet as described in detail in Snyder et al. (2006a). With respect to SNPs at codon 16 of the ADRB2, the primary objective of visit 3 for this study was to compare responses pertaining to *V̇*_A_ and alveolar and arterial O_2_ tensions with respect to metabolic demand. This was accomplished by having participants perform 18 consecutive minutes of submaximal cycle ergometry at two separate blocked workloads set at 40% and 75% of peak workload (determined from CPET).

### Data collection

#### Genotyping

A complete description of the protocol used to genotype codon 16 of the ADRB2 using the polymerase chain reaction (PCR) method is presented in Snyder et al. (2006a) and based on techniques of Bray et al. (2000). Therefore, in brief, the following primer sequences used, forward and backward, were 5'‐AGC CAG TGC GCT TAC CTG CCA GAC‐3' (at –32) and 3'‐CA TGG GTA CGC GGC CTG GTG CTG CAG TGC –5', respectively. This resulted in a PCR product 107 base‐pairs in length. As such, the Arg16Arg genotype is represented by a single 107 base‐pair band; the Arg16Gly genotype is represented by 25‐, 82‐, and 107 base‐pair bands; and the Gly16Gly genotype is represented by 82‐ and‐ 260 base‐pair bands.

#### Pulmonary function

Resting pulmonary function was assessed using flow‐volume loop spirometry (CPFS system spirometer, Medical Graphics, St. Paul, MN) in the upright seated position according to guidelines of the American Thoracic Society (Miller et al. 2005). In addition to measuring forced vital capacity (FVC) and forced expiratory volume in 1 sec (FEV_1_), percent of predicted FVC and FEV_1_ were calculated according to equations of Crapo et al. (1981). We calculated maximum voluntary ventilation (MVV) as the product of FEV_1_ and 40 (Miller et al. 2005).

#### Exercise testing

Participants were studied in the postabsorptive state and absence of caffeine ingestion. With continuous rhythm and heart rate monitoring via 12‐lead electrocardiogram, participants performed a step‐wise CPET to volitional fatigue via upright cycle ergometry (Corival Lode B.V., Netherlands). Testing began with a 3 min rest period followed immediately by a 3 min exercise workload period set at 40 W, increasing thereafter in 40 W increments every 3 min until volitional fatigue (American Thoracic S, and American College of Chest P, 2003; Van Iterson et al. 2017c). Participants were asked to maintain a pedal cadence of 60–65 rpm throughout CPET. An inability of participants to maintain a pedal cadence of 60–65 rpm, a rating of perceived exertion (RPE, Borg 6–20 scale) at the end of an exercise stage ≥17, and/or respiratory exchange ratio (RER) ≥1.10 were closely monitored throughout CPET and were used to assess when peak exercise was achieved (American Thoracic S, and American College of Chest P, 2003; Van Iterson et al. 2017c). Percent of predicted *V̇*O_2peak_ was calculated using equations of Hansen et al. (1984).

Submaximal cycle ergometry performed on study visit 3 was performed for 18 consecutive min at a pedal cadence of 60–65 rpm and relative workload intensities equivalent to 40% and 75% of peak workload determined from CPET. Following an initial rest period of 3 min, participants transitioned to exercise at 40% of peak workload for 9 consecutive min immediately transitioning thereafter to a workload equivalent of 75% of peak workload for 9 more min. In addition to continuous breath‐by‐breath measurements of ventilation and gas exchange throughout exercise, arterial draws were performed during steady‐state exercise to assess blood gases (described below).

#### Ventilation and gas exchange measurements

Standard breath‐by‐breath measurements of ventilation (minute ventilation [*V̇*_E_]), volumes (tidal volume [*V*_T_]), and gas exchange (*V̇*O_2_ and carbon dioxide output [*V̇*CO_2_]) variables occurred continuously throughout all exercise testing in an environmentally controlled human physiological laboratory (FIO_2 _= 0.2093 ± 0.0001; room temperature did not fluctuate more than ± 1°C from 21°C). These variables were acquired using an open circuit indirect calorimetry system (Medical Graphics, St. Paul, MN) customized to sample respired gas fractions in alignment with volume flows via custom software integrated with gas mass spectroscopy (Perkin Elmer MGA‐1100, Wesley, MA). Sampling of respired gas fractions using this system has been validated in our laboratory against the Douglas bag technique (Proctor and Beck 1996). Relevant for study visit 3, data from respired gas fractions and arterial gases were used in “ideal” alveolar air equations (Riley and Cournand 1951; Van Iterson et al. 2017b) for the calculation of *V̇*_A_ and related variables (see Appendix 1). Calibration of the system using medical grade gases and linearity of the system flowmeter via 3 L syringe across a range of flows was performed using standard routines in the set‐up used for testing immediately prior to each exercise test.

#### Arterial sampling

For calculations relevant to *V̇*_A_ [i.e., “ideal” alveolar air equations (Riley and Cournand 1951; Van Iterson et al. 2017b), see Appendix 1] arterial draws were temporally aligned with the 30 sec averaged periods for variables of interest at rest as well as near the end of each 3 min interval throughout submaximal exercise on study visit 3. Temporal alignment of non‐invasive and invasive data in this manner is suggested to be accurate during steady‐state exercise (Furuike et al. 1982). Accordingly, using standard technique at the left radial artery, percutaneous insertion of a 20‐gauge indwelling catheter (Arrow International, Reading, PA) with thermistor was used to draw arterial samples. Arterial samples were drawn into 3 mL heparinized glass syringes and immediately rolled and placed in ice to be transported to the Mayo Clinic institutional Clinical Core Laboratory [meets all routine standards of clinical blood‐gas laboratory (Davis et al. 2013)] for measurements of CO_2_ tension (PaCO_2_), O_2_ tension (PaO_2_), and Hb oxygen saturation (SaO_2_). We used the equation, (0.0134 × Hb×SaO_2_) + (0.0031 × PaO_2_), to calculate CaO_2_. There were no between group differences for inspired tension of O_2_ (PIO_2_) on study visit 3 (143 ± 2, 142 ± 1, and 143 ± 1 mmHg for Arg16Arg, Arg16Gly, and Gly16Gly, respectively, *P *>* *0.05).

#### Metabolic computations

Steady‐state mean values for *V̇*O_2_ and *V̇*CO_2_ representing the final 30 sec of both low (40%) and moderate (75%) intensity exercise periods were used to compute gross metabolic demand as nonprotein substrate oxidation (Brouwer 1957; Coyle et al. 1992; Moseley and Jeukendrup 2001). As such, we quantified exercise economy (EC, i.e., a lower value is worse) as the ratio of work accomplished per L/(min·m^2^) of *V̇*O_2_ expressed in units of kcal/(L·m^2^) as (Moseley and Jeukendrup 2001): $\text{workload}÷\dot{V}O_{2}$ where workload is W converted to kcal/min. We also quantified net EC (EC_NET_, i.e., a higher value is worse) as the absolute difference between energy expended (EE) and work accomplished per L/(min·m^2^) of *V̇*O_2_ expressed in units of kcal/(L·m^2^). We calculated EE in units of kcal/min as in Brouwer (1957):

$$\begin{array}{c} \left(\left[\left(\dot{V}O_{2}\times 3.869\right)+\left(\dot{V}{\text{CO}}_{2}\times 1.195\right)\times \left(4.186÷60\right)\times 1000\times 4.2÷1000\right]\times 60\right) \end{array}$$

### Statistical analyses

Data are presented as mean ± SD with 95% confidence limits (CL) where appropriate. All data met assumptions of normality of distribution and homogeneity of variance. The group effect for demographic data was assessed using single‐factor ANOVA or Kruskal–Wallis tests with post hoc testing performed using the Tukey–Kramer or Wilcoxon rank sum test, respectively, to identify pairwise differences when the overall group effect was significant.

Data reported and used for statistical analyses with respect to submaximal exercise variables is reflective of steady‐state mean values taken from the final 30 s of the low (40%) and moderate (75%) intensity exercise periods. Between group differences were assessed using repeated measures single‐factor ANOVA tests. Only when the *F*‐test statistic was significant from ANOVA testing did we assess planned pairwise differences using the Tukey–Kramer post hoc test. Where applicable, least squares univariate linear regression models were used to assess the behavior of physiological relationships for *β*_2_AR genotypes [e.g., between *V̇*_E_ (independent) and *V̇*_A_ (dependent)]. Two‐tailed significance was determined using an alpha level set at 0.05. All computations were performed using SAS statistical software (v.9.4., Cary, North Carolina).

## Results

### Participants

Table 1 illustrates there was no overall group effect for gender, age, height, weight, BMI, BSA, Hb, or Hct. There was also no overall group effect for MVV and resting measurements of absolute or percent of predicted FVC or FEV_1_. All participants reached peak exercise during CPET indicated by both RER and RPE (Table 1). Although the group effect was not significant for peak exercise workload, the Arg16Gly group achieved the highest W. However, except for *V*_T_, there was no overall group effect for *V̇*O_2_ (both L/(min·m^2^) and percent of predicted), heart rate, *V̇*_E_, %MVV, or breathing frequency (*f*_B_) associated with baseline CPET.

**Table 1:** Participant characteristics

	Arg16Arg (*N* = 16)	Arg16Gly (*N* = 17)	Gly16Gly (*N* = 31)	*P*
% male	44	47	52	0.88
Age, years	29 ± 6 (26, 32)	28 ± 6 (25, 31)	29 ± 6 (27, 32)	0.85
Height, cm	171 ± 9 (167, 176)	176 ± 10 (171, 181)	174 ± 10 (170, 178)	0.41
Weight, kg	67 ± 12 (61, 73)	76 ± 14 (67, 82)	75 ± 13 (70, 80)	0.14
BMI, kg/m^2^	23 ± 3 (21, 24)	24 ± 3 (22, 25)	25 ± 4 (23, 26)	0.21
BSA, m^2^	1.79 ± 0.05 (1.68, 1.87)	1.91 ± 0.05 (1.78, 2.01)	1.89 ± 0.04 (1.81, 1.97)	0.16
Hemoglobin, g/dL	13.4 ± 1.3 (12.6, 13.9)	13.7 ± 1.1 (13.1, 14.3)	13.7 ± 1.1 (13.2, 14.1)	0.48
Hematocrit, %	39 ± 3 (37, 40)	40 ± 3 (38, 41)	40 ± 3 (38, 42)	0.45
Resting Pulmonary function				
FVC, L	4.5 ± 0.9 (4.0, 5.0)	5.2 ± 1.2 (4.6, 5.9)	5.0 ± 1.1 (4.6, 5.4)	0.16
FVC, %pred.	99 ± 13 (92, 105)	105 ± 9 (100, 110)	102 ± 9 (98, 105)	0.25
FEV_1_, L	3.7 ± 0.7 (3.4, 4.1)	4.1 ± 0.8 (3.7, 4.6)	4.2 ± 0.9 (3.8, 4.5)	0.21
FEV_1_, %pred.	99 ± 13 (92, 105)	100 ± 9 (95, 105)	103 ± 10 (99, 107)	0.35
MVV, L/min	149 ± 27 (135, 163)	165 ± 34 (148, 183)	166 ± 37 (152, 180)	0.21
Peak exercise				
*V̇*O_2_, L/(min·m^2^)	1.2 ± 0.3 (1.1, 1.4)	1.4 ± 0.3 (1.3, 1.6)	1.4 ± 0.3 (1.3, 1.5)	0.11
*V̇*O_2_, %pred.	86 ± 23 (74, 98)	98 ± 29 (82, 114)	91 ± 22 (82, 100)	0.41
Workload, W	185 ± 56 (156, 214)	244 ± 64 (209, 278)	224 ± 78 (194, 254)	0.06
HR, bpm	189 ± 10 (184, 194)	182 ± 9 (177, 187)	188 ± 10 (184, 192)	0.07
*V̇*_E_, L/min	87 ± 31 (71, 103)	100 ± 25 (87, 114)	102 ± 31 (90, 113)	0.24
*V̇*_E_, %MVV	57 ± 12 (51, 63)	61 ± 11 (55, 67)	61 ± 12 (57, 66)	0.49
*f*_B_, breaths/min	44 ± 9 (39, 48)	41 ± 7 (37, 45)	40 ± 7 (37, 43)	0.31
*V*_T_, L	1.98 ± 0.56 (1.69, 2.26)	2.50 ± 0.66 (2.15, 2.85)	2.56 ± 0.73 (2.29, 2.84)^a^	0.02
RER	1.17 ± 0.06 (1.13, 1.20)	1.15 ± 0.05 (1.12, 0.17)	1.15 ± 0.06 (1.13, 1.18)	0.44
RPE	19 ± 0 (19, 19)	19 ± 0 (19, 19)	19 ± 0 (19, 19)	0.07
Submaximal exercise workload and energy expended				
Low, W	70 ± 20 (60, 81)	86 ± 22 (74, 98)	84 ± 28 (73, 94)	
EE, kcal/min	5.7 ± 1.7 (4.9, 6.6)	6.5 ± 1.4 (5.8, 7.2)	6.4 ± 1.6 (5.8, 7.1)	
Moderate, W	139 ± 43 (116, 163)	168 ± 45 (144, 192)	169 ± 60 (145, 192)	
EE, kcal/min	11.0 ± 3.5 (9.1, 12.8)	13.0 ± 3.2 (11.3, 14.7)	13.7 ± 4.7 (11.8, 15.5)^a^	

Data are mean ± SD and 95% lower and upper confidence limits (CL), or otherwise noted. FVC, forced vital capacity; FEV_1_, forced expiratory volume in 1 sec; MVV, maximum voluntary ventilation; *V̇*O_2_, pulmonary O_2_ uptake; HR, heart rate; *V̇*_E_, minute ventilation; *f*_B_, breathing frequency; *V*_T_, tidal volume; RER, respiratory exchange ratio; RPE, rating of perceived exertion (Borg, 6–20 scale); EE, energy expended. Repeated measures ANOVA for group effect on submaximal exercise workload, *F*[91], *P *<* *0.001; there were no pairwise differences at an alpha level of 0.05 after Tukey–Kramer post hoc correction. Test‐retest reliability of our CPET was strong [Intraclass correlation coefficient across the sample for peak workload between study 1 and 2 was 0.98 with lower and upper 95% CL: 0.95, 0.99]. *P*‐values in table are overall group effect from ANOVA testing.

a *P *<* *0.05, Arg16Arg versus Gly16Gly after Tukey–Kramer post hoc correction.

### Submaximal exercise testing

**Figure 1:**
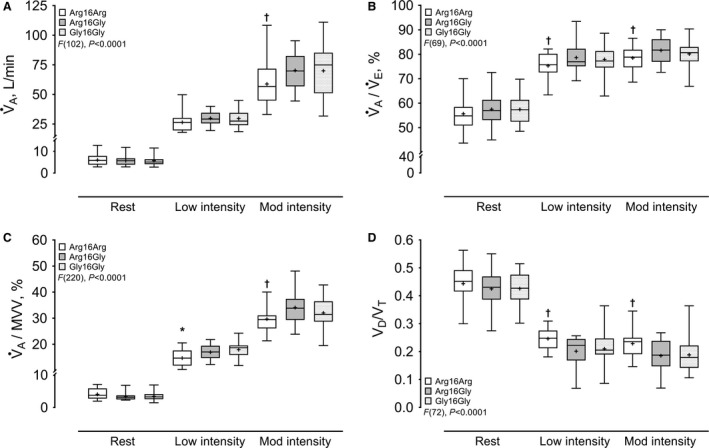
Respiratory responses to low (40% peak workload) and moderate (75% peak workload) intensity exercise in healthy adults stratified by SNPs at codon 16 of ADRB2. *N* = 16, homozygous for amino acid arginine (Arg16Arg); *N* = 17, heterozygous for arginine and glycine (Arg16Gly); *N* = 31, homozygous for glycine (Gly16Gly). Data are interquartile range with the group means indicated by (+). (A) alveolar ventilation, *V̇*_A_; (B) V̇_A_ as a percentage of total minute ventilation, *V̇*_A_/*V̇*_E_; (C) *V̇*_A_ as a percentage of maximum voluntary ventilation *V̇*_A_/MVV; (D) Physiological dead space to tidal volume ratio, *V*_D_/*V*_T_. *P <* *0.05, Arg16Arg versus Gly16Gly; †*P *<* *0.05, Arg16Arg versus both Arg16Gly and Gly16Gly. Significance following Tukey–Kramer post hoc correction.

**Figure 2:**
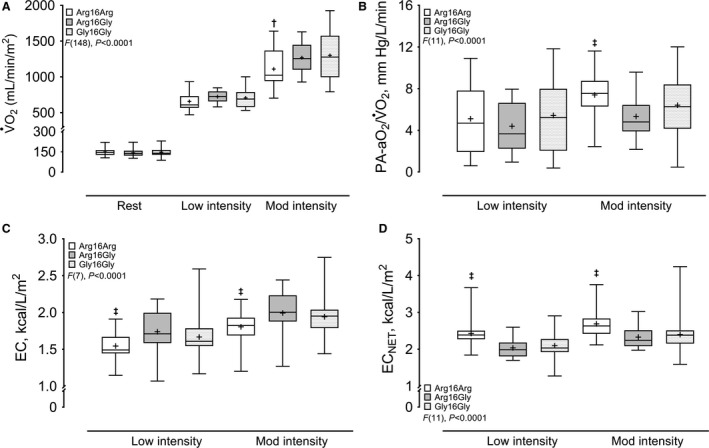
Oxygen uptake and gross metabolic demand during low (40% peak workload) and moderate (75% peak workload) intensity exercise in healthy adults stratified by SNPs at codon 16 of the ADRB2. *N* = 16, homozygous for amino acid arginine (Arg16Arg); *N* = 17, heterozygous for arginine and glycine (Arg16Gly); *N* = 31, homozygous for glycine (Gly16Gly). Data are interquartile range with the group means indicated by (+). A) pulmonary O_2_ uptake, *V̇*O_2_; (B) quotient of alveolar‐to‐arterial O_2_ tension gradient with *V̇*O_2_, PA‐aO_2_/*V̇*O_2_; (C) exercise economy, EC; (D) net exercise economy, EC_NET_. †*P *<* *0.05, Arg16Arg versus both Arg16Gly and Gly16Gly; ‡*P *<* *0.05, Arg16Arg versus Arg16Gly. Significance following Tukey–Kramer post hoc correction.

Although there were no pairwise statistical differences for exercise workload, at both low and moderate intensity Arg16Arg demonstrated the lowest W (Table 1). In contrast, there was a group effect for gross metabolic demand (i.e., nonprotein substrate oxidation during exercise) (*F*[30], *P *<* *0.001), which resulted in significantly lower absolute EE in Arg16Arg compared with Gly16Gly during moderate intensity, but not at low intensity (Table 1). Overall, there were also significant group effects for *β*_2_AR genotype on *V̇*_A_, *V̇*_A_ as a percentage of *V̇*_E_ (*V̇*_A_/*V̇*_E_), *V̇*_A_ as a percentage of MVV (V̇_A_/MVV), and physiological dead space to tidal volume ratio (*V*_D_/*V*_T_) in Figure 1; *V̇*O_2_, alveolar‐to‐arterial O_2_ tension difference (PA‐aO_2_) as a quotient with *V̇*O_2_ (PA‐aO_2_/V̇O_2_), EC, and EC_NET_ in Figure 2; and *V̇*_E_, *f*_B_, *V*_T_, *V*_A_, *V*_A_ as percentage of resting FVC (*V*_A_/FVC), alveolar O_2_ tension (PAO_2_), alveolar CO_2_ tension (PACO_2_), PaCO_2_, CaO_2_, PA‐aO_2_, and SaO_2_ in Table 2 (but not for PaO_2_).

**Table 2:** Basic ventilation, alveolar air, and arterial blood responses across genotypes for the β_2_AR

	Arg16Arg (*N* = 16)	Arg16Gly (*N* = 17)	Gly16Gly (*N* = 31)
Rest			
*V̇*_E_, L/min	11 ± 4 (8, 13)	10 ± 3 (8, 11)	10 ± 4 (8, 11)
*f*_B_, breaths/min	16 ± 4 (13, 18)	15 ± 5 (12, 17)	14 ± 3 (13, 15)
*V*_T_, L	0.74 ± 0.44 (0.51, 0.97)	0.75 ± 0.32 (0.55, 0.95)	0.72 ± 0.39 (0.61, 0.84)
*V*_A_, L	0.43 ± 0.28 (0.28, 0.57)	0.45 ± 0.30 (0.29, 0.61)	0.42 ± 0.20 (0.34, 0.50)
*V*_A_/FVC, %	9.6 ± 6.2 (6.4, 12.8)	8.6 ± 5.0 (6.0, 11.3)	8.4 ± 3.2 (7.2, 9.7)
PAO_2_, mmHg	101 ± 11 (95, 106)	98 ± 8 (94, 103)	98 ± 7 (95, 101)
PaO_2_, mmHg	96 ± 14 (90, 104)	95 ± 8 (91, 100)	95 ± 11 (91, 99)
PACO_2_, mmHg	33 ± 5 (31, 35)	34 ± 3 (32, 36)	34 ± 3 (33, 35)
PaCO_2_, mmHg	34 ± 5 (32, 37)	36 ± 4 (33, 38)	36 ± 4 (34, 37)
CaO_2_, mL/dL	18.6 ± 1.9 (17.7, 19.6)	19.4 ± 1.7 (18.5, 20.3)	19.4 ± 1.9 (18.7, 20.1)
PA‐aO_2_, mmHg	6 ± 3 (4, 8)	4 ± 3 (2, 5)	5 ± 4 (4, 7)
SaO_2_, %	98 ± 1 (97, 98)	98 ± 0 (97, 98)	98 ± 1 (97, 98)
Low intensity exercise			
*V̇*_E_, L/min	35 ± 9 (30, 39)	37 ± 7 (34 41)	37 ± 8 (35, 40)
*f*_B_, breaths/min	26 ± 6 (23, 29)	26 ± 5 (23, 29)	25 ± 5 (23, 27)
*V*_T_, L	1.37 ± 0.40 (1.17, 1.58)	1.50 ± 0.44 (1.27, 1.73)	1.60 ± 0.49 (1.41, 1.78)
*V*_A_, L	1.04 ± 0.30 (0.88, 1.19)^a^	1.21 ± 0.43 (0.98, 1.44)	1.28 ± 0.46 (1.10, 1.45)
*V*_A_/FVC, %	22.0 ± 5.3 (19.2, 24.7)^a^	23.6 ± 5.9 (20.4, 26.7)	25.3 ± 6.0 (23.0, 27.5)
PAO_2_, mmHg	104 ± 5 (101, 106)	103 ± 3 (100, 104)	104 ± 4 (102, 105)
PaO_2_, mmHg	99 ± 5 (96, 101)	99 ± 8 (94, 103)	98 ± 6 (96, 100)
PACO_2_, mmHg	36 ± 3 (35, 38)	36 ± 3 (34, 37)	36 ± 3 (35, 37)
PaCO_2_, mmHg	37 ± 3 (35, 38)	37 ± 3 (35, 38)	36 ± 3 (35, 37)
CaO_2_, mL/dL	19.2 ± 1.8 (18.2, 20.1)	20.0 ± 1.8 (19.0, 21.0)	20.2 ± 1.8 (19.6, 20.9)
PA‐aO_2_, mmHg	6 ± 4 (4, 8)	6 ± 3 (4, 8)	7 ± 5 (5, 10)
SaO_2_, %	98 ± 1 (97, 98)	97 ± 0 (97, 97)	97 ± 1 (97, 98)
Moderate intensity exercise			
*V̇*_E_, L/min	75 ± 26 (61, 90)	86 ± 19 (76, 97)	87 ± 27 (76, 97)
*f*_B_, breaths/min	40 ± 7 (36, 44)	38 ± 6 (34, 41)	37 ± 8 (34, 41)
*V*_T_, L	1.85 ± 0.48 (1.59, 2.10)^b^	2.37 ± 0.66 (2.02, 2.72)	2.38 ± 0.75 (2.08, 2.67)
*V*_A_, L	1.43 ± 0.37 (1.24, 1.62)^b^	1.95 ± 0.61 (1.62, 2.28)	1.93 ± 0.65 (1.68, 2.19)
*V*_A_/FVC, %	32.0 ± 4.7 (29.5, 34.5)^b^	36.9 ± 6.4 (33.5, 40.3)	38.0 ± 7.7 (35.0, 41.1)
PAO_2_, mmHg	113 ± 4 (111, 115)	112 ± 4 (110, 114)	112 ± 4 (110, 113)
PaO_2_, mmHg	101 ± 8 (96, 105)	101 ± 10 (96, 106)	98 ± 9 (95, 102)
PACO_2_, mmHg	31 ± 4 (30, 33)	31 ± 3 (29, 32)	32 ± 5 (30, 34)
PaCO_2_, mmHg	31 ± 4 (29, 33)	31 ± 3 (28, 32)	32 ± 3 (30, 33)
CaO_2_, mL/dL	19.9 ± 1.9 (18.9, 20.9)	21.0 ± 1.8 (20.0, 22.0)	20.7 ± 1.9 (20.0, 21.4)
PA‐aO_2_, mmHg	14 ± 5 (11, 17)	12 ± 5 (10, 15)	16 ± 8 (13, 19)
SaO_2_, %	97 ± 1 (96, 97)	97 ± 0 (96, 97)	97 ± 1 (96, 97)

Data are mean ± SD with lower and upper 95% confidence limits (CL) in parentheses. Low (40% peak workload) or moderate (75% peak workload) intensity exercise. *F*‐statistic from ANOVA for: minute ventilation (*V̇*_E_, *F*[128], *P *<* *0.0001); breathing frequency (*f*_B_, *F*[77], *P *<* *0.0001); tidal volume (*V*_T_, *F*[54], *P *<* *0.0001); alveolar volume (*V*_A_, *F*[58], *P *<* *0.0001); V_A_ as percentage resting forced vital capacity (*V*_A_/FVC, *F*[80], *P *<* *0.0001); alveolar O_2_ tension (PAO_2_, *F*[54], *P *<* *0.0001); arterial O_2_ tension (PaO_2_, *F*[1.3], *P *=* *0.28); alveolar CO_2_ tension (PACO_2_, *F*[12], *P *<* *0.0001); arterial CO_2_ tension (PaCO_2_, *F*[25], *P *<* *0.0001); arterial O_2_ content (CaO_2_, *F*[48], *P *<* *0.0001); alveolar‐to‐arterial O_2_ difference (PA‐aO_2_, *F*[8], *P *<* *0.0001); and arterial saturation (SaO_2_, *F*[4.5], *P *<* *0.0001).

a *P *<* *0.05, Arg16Arg versus Gly16Gly.

b *P *<* *0.05, Arg16Arg versus both Arg16Gly and Gly16Gly. Significance following Tukey–Kramer post hoc correction.

#### Low intensity

There were no pairwise differences for any variable at rest. In contrast, during low intensity exercise at 40% of peak workload, Arg16Arg demonstrated significantly smaller *V*_A_ and *V*_A_/FVC compared with Gly16Gly, whereas *V*_T_ and CaO_2_ trended (*P *=* *0.10 and *P *=* *0.06) lower in Arg16Arg versus Gly16Gly (Table 2). Arg16Arg also demonstrated significantly reduced *V*_A_/MVV in comparison with Gly16Gly (Fig. 1C), whereas *V*_D_/*V*_T_ was significantly larger in Arg16Arg compared with both Arg16Gly and Gly16Gly (Fig. 1D). Figure 1B also illustrates *V*_A_/*V*_E_ was significantly reduced in Arg16Arg compared with both Arg16Gly and Gly16Gly. Figure 1A shows *V*_A_ trended lower in Arg16Arg in comparison with both Arg16Gly and Gly16Gly (*P *=* *0.11 and *P *=* *0.09, respectively). Likewise, though *V*O_2_ trended lower in Arg16Arg versus Arg16Gly in Figure 2A (*P *=* *0.12), EC and EC_NET_ were significantly reduced and increased, respectively, in Arg16Arg compared with Arg16Gly in Figure 2 (panels C and D). Whereas, similar *V̇*O_2_ in Arg16Arg and Gly16Gly was accompanied by a pattern of decreased and increased EC and EC_NET_, respectively, between Arg16Arg (*P *=* *0.10) and Gly16Gly (*P *=* *0.11) (Fig. 2). There were no pairwise group differences for the remaining variables in Table 2 or Figures 1 and 2 at low intensity exercise.

**Figure 3:**
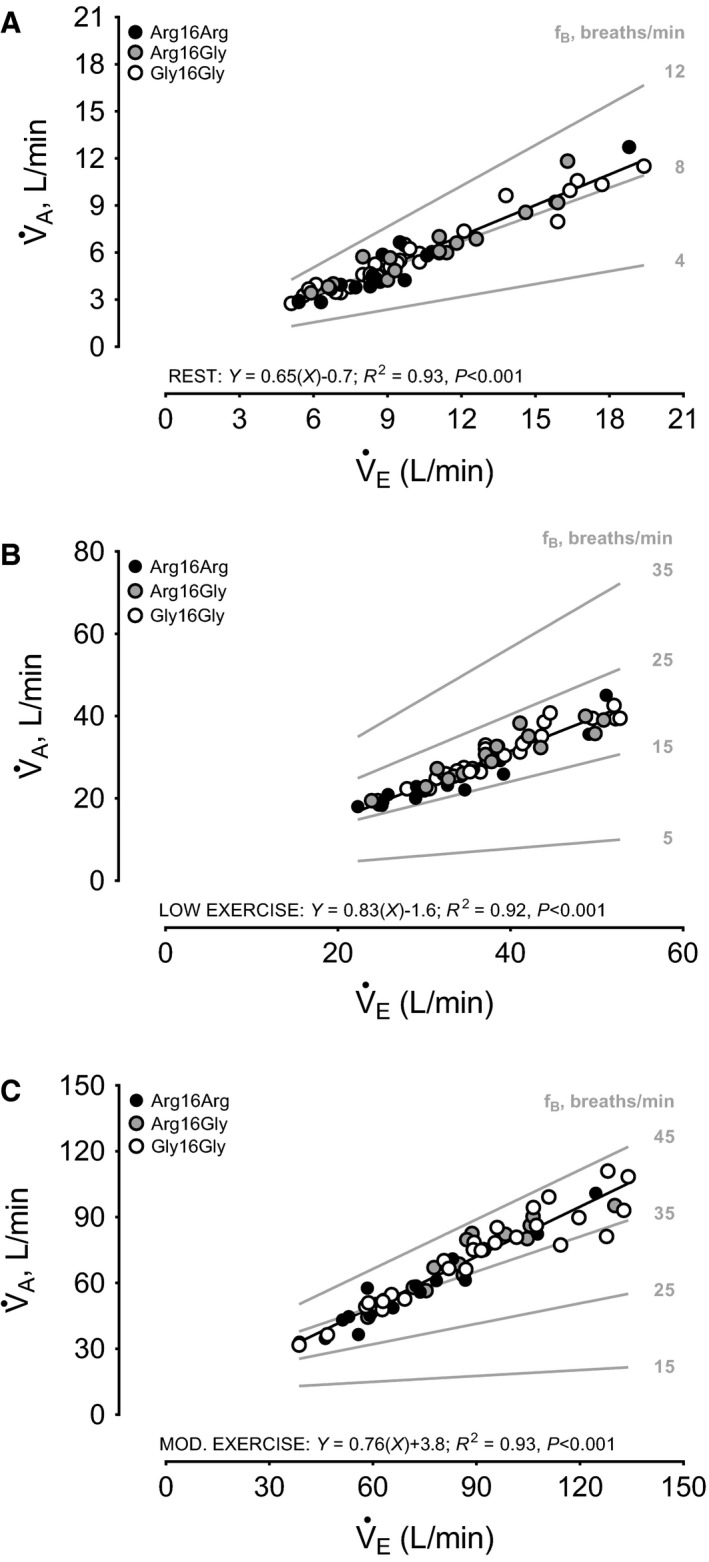
Least squares univariate linear regression between total minute ventilation (*V̇*_E_) (independent) and alveolar ventilation (*V̇*_A_) (dependent) during low (40% peak workload) and moderate (75% peak workload) intensity exercise in healthy adults stratified by SNPs at codon 16 of the ADRB2. *N* = 16, homozygous for amino acid arginine (Arg16Arg); *N* = 17, heterozygous for arginine and glycine (Arg16Gly); *N* = 31, homozygous for glycine (Gly16Gly). Solid black line is model goodness of fit for the regression across the entire sample. Grey lines are isopleths representing the expected *V̇*_A_ response for a given observed *V̇*_E_ response when breathing frequency (*f*_B_) is constrained (grey numbers within plots) for a given observed alveolar volume (*V*_A_) response. (A) Rest: Arg16Arg, *Y* = 0.67(*X*)‐1.1, *P *<* *0.001; Arg16Gly, *Y* = 0.69(*X*)‐1.0, *P *<* *0.001; Gly16Gly, *Y* = 0.63(*X*)‐0.5, *P *<* *0.001. (B) 40% peak workload: Arg16Arg, *Y* = 0.74(*X*) + 0.5, *P *<* *0.001; Arg16Gly, *Y* = 0.85(*X*)‐1.7, *P *<* *0.001; Gly16Gly, *Y* = 0.85(*X*)‐2.5, *P *<* *0.001. (C) 75% peak workload: Arg16Arg, *Y* = 0.74(*X*) + 2.6, *P *<* *0.001; Arg16Gly, *Y* = 0.88(*X*)‐3.9, *P *<* *0.001; Gly16Gly, *Y* = 0.82(*X*) + 0.3, *P *<* *0.001.

Consistent with rest in Figure 3A, the relationship (coefficient of determination, *R* ^2^) between *V̇*_E_ (independent) and *V̇*_A_ (dependent) during low intensity exercise was significant across the entire sample in Figure 3B. Likewise, individual *R* ^2^ for these relationships were equally strong for Arg16Arg (*R* ^2 ^= 0.96, *P *<* *0.001), Arg16Gly (*R* ^2^=0.93, *P *<* *0.001), and Gly16Gly (*R* ^2^=0.94, *P *<* *0.001). However, consistent with reduced V_A_ and V_A_/FVC for Arg16Arg in Table 2, the extended response of *V*_A_ in driving further increases in *V̇*_A_ beyond contributions from *f*_B_ was not as strong for Arg16Arg compared with both Arg16Gly and Gly16Gly [standardized *β* with *95%* CL (i.e., slope) for *V*_A_→*V̇*_A_ relationships were: 0.57 (0.11, 0.82), 0.74 (0.37, 0.90), and 0.78 (0.58, 0.89), respectively]. This is also illustrated in gray isopleths as progressively steeper slopes for *V̇*_E_→*V̇*_A_ relationships when we constrained *f*_B_ at modest‐to‐moderate levels (15 and 25 breaths/min) (Fig. 3B).

#### Moderate intensity

For exercise at 75% of peak workload, Arg16Arg demonstrated significantly lower *V̇*_A_ (Fig. 1A), *V*_T_, *V*_A_, and *V*_A_/FVC compared with both Arg16Gly and Gly16Gly (Table 2). Likewise, consistent with significant pairwise differences for *V*_A_/*V̇*_E_ and *V̇*_A_/MVV (Fig. 1, panels B and C, respectively), Figure 1D illustrates *V*_D_/*V*_T_ was significantly larger in Arg16Arg compared with both Arg16Gly and Gly16Gly. This was accompanied by significantly reduced *V̇*O_2_ in Arg16Arg compared with both Arg16Gly and Gly16Gly in Figure 2A. In contrast, PA‐aO_2_/*V̇*O_2_ was significantly increased for Arg16Arg compared with Arg16Gly in Figure 2B, but did not differ when Arg16Arg was compared with Gly16Gly (*P *=* *0.43). While CaO_2_ also did not differ significantly across groups, there was a pattern for lower values in Arg16Arg versus Arg16Gly or Gly16Gly (*P *=* *0.11 and *P *=* *0.15, respectively; Table 2). However, consistent with differences at low intensity exercise, EC and EC_NET_ were significantly reduced and increased, respectively, for Arg16Arg compared with Arg16Gly in Figure 2, whereas these variables did not differ between Arg16Arg versus Gly16Gly (*P *=* *0.29 and *P *=* *0.24, respectively). There were no other pairwise group differences for variables presented in Table 2 or Figures 1 and 2 for moderate intensity exercise.

The strength of the relationship between *V̇*_E_ (independent) and *V̇*_A_ (dependent) across the entire sample at low intensity exercise in Figure 3B persisted to moderate intensity exercise in Figure 3C. Individual *R* ^2^ between *V̇*_E_ and *V̇*_A_ were also strong for Arg16Arg (*R* ^2 ^= 0.94, *P *<* *0.001), Arg16Gly (*R* ^2 ^= 0.94, *P *<* *0.001), and Gly16Gly (*R* ^2 ^= 0.93, *P *<* *0.001). However, consistent with relationships in Figure 3B and absolute values in Table 2, the blunted contribution of *V*_A_ to the *V̇*_E_→*V̇*_A_ relationship when *f*_B_ was constrained at moderate‐to‐high levels (i.e., gray isopleths at 35 and 45 breaths/min, respectively) was indeed more depressed with increasing *V̇*_E_ for Arg16Arg in comparison with both Arg16Gly and Gly16Gly (Figure 3C). This was consistent with the standardized *β* (95% CL) (i.e., slopes) for individual *V*_A_→*V̇*_A_ relationships for Arg16Arg compared with both Arg16Gly and Gly16Gly [0.53 (0.05, 0.80), 0.83 (0.54, 0.94), and 0.83 (0.65, 0.91), respectively].

## Discussion

These data suggest that during low‐to‐moderate intensity aerobic exercise and for a given *V̇*_E_, healthy adults demonstrating the Arg16Arg SNP for the ADRB2 display a blunted rise in *V̇*_A_ attributable to disproportionately small *V*_A_ relative to *f*_B_. Compared with both Arg16Gly and Gly16Gly *β*_2_AR genotypes, Arg16Arg likewise demonstrated consistently larger *V*_D_/*V*_T_ throughout exercise, whereas the most prominent rise in PA‐aO_2_/*V̇*O_2_ occurred during the moderate intensity period. While we did not, and were not expecting to observe severe or even moderate exercise induced arterial hypoxemia (SaO_2_, <88% or 88–93%, respectively) given the present workload intensities coupled with an absence of cardiopulmonary disease, it is still consistent with these data that relative to each group a modest‐to‐moderate pattern of decreased CaO_2_ occurred for adults demonstrating the Arg16Arg *β*_2_AR genotype. In this context, and as hypothesis generating observations, these data suggest integrated responses of *V*_A_ (both absolute and relative to FVC), *V̇*_A_ (both absolute and relative to *V̇*_E_), *V*_D_/*V*_T_, PA‐aO_2_/*V̇*O_2_ (as a broad surrogate of lung diffusing capacity for O_2_) (Morosin et al. 2016), and CaO_2_ collectively trended in a direction consistent with supporting our study hypothesis. Though we also acknowledge that it cannot be unequivocally concluded based on our isolated genomics studies that the Arg16Arg SNP of the ADRB2 is fully responsible for the present physiological observations, these results further indicate that given the present study paradigm, compared with Arg16Gly and Gly16Gly *β*_2_AR genotypes, Arg16Arg healthy adults do not demonstrate a similar capacity to drive *V*_A_ and *V̇*_A_ relative to substrate oxidative capacity and exercise economy.

Independent of our proposed effects of abnormal alveolar respiration, the inability to economically meet metabolic demands of submaximal exercise in Arg16Arg compared with Arg16Gly and Gly16Gly *β*_2_AR genotypes in this study is broadly consistent with reports suggesting ~99% of β‐adrenergic receptors in skeletal muscle are *β*_2_ARs (Liggett et al. 1988) and in muscle diseases such as Myasthenia Gravis, there is an increased likelihood for patients demonstrating the Arg16Arg genotype (Xu et al. 2000). Accordingly, while those and other studies of skeletal muscle phenotypes and *β*_2_ARs might be taken to imply limited oxidative capacity associated with the Arg16Arg variant may be directly attributable to skeletal muscle origins (Liggett et al. 1988; Xu et al. 2000; Wolfarth et al. 2007), this SNP for the ADRB2 has also been separately linked to reduced *Q̇* (attributed to blunted increases in stroke volume), *β*_2_AR desensitization followed by increased vascular resistance, and decreased airway function at rest and/or during exercise in healthy adults (Dishy et al. 2001; Garovic et al. 2003; Snyder et al. 2006a,b). This suggests that although skeletal muscle factors contribute to changes in oxidative capacity, which perhaps may or may not be underpinned by SNPs of the ADRB2 (Liggett et al. 1988; Xu et al. 2000; Wolfarth et al. 2007), it is also likely that *β*_2_AR expression and function involving the whole body O_2_ transport chain including cardiac, smooth, and skeletal muscle is of consequence to exercise capacity (Kjaer et al. 1985; Garovic et al. 2003; Snyder et al. 2006a,b).

Therefore, because in the lung there is a predominating expression of *β*_2_ARs on alveolar tissue coupled with the role these receptors play in helping to maintain the alveolar surface area needed for gas exchange, for the first time, this study sought to assess in what manner might SNPs of the ADRB2 translate to coupled alveolar respiratory and metabolic responses to submaximal exercise in healthy adults. Though it is known *β*_2_ARs are not directly responsible for facilitating the transfer of O_2_ across the alveolar‐capillary membrane, functional receptors expressed on alveolar tissue are critically needed for proper gas exchange required during exercise and/or stays in extreme environments (e.g., high altitude pulmonary edema) (Kerem et al. 1999; Crandall and Matthay 2001; McGraw et al. 2001; Sartori et al. 2002; Snyder et al. 2006d, 2007).

In this study, the period of moderate exercise performed by participants, despite not being of maximal intensity, has been reported by others as being an adequate stimulus for provoking modest‐to‐moderate lung fluid accumulation in some, but not all healthy adults (Coates et al. 1984; Koizumi et al. 2001; McKenzie et al. 2005; Snyder et al. 2006c). As such, while we hypothesize that contrasting alveolar respiratory responses during exercise in Arg16Arg compared with Arg16Gly and Gly16Gly variants in this study may have been attributable to abnormal alveolar *β*_2_AR function and reduced total alveolar surface area in the former, we did not directly assess receptor function/density or measure lung fluid changes during exercise and thereby cannot confirm this genotype↔phenotype mechanism as the explanation for our observations. However, because the capacity to recruit *V*_T_ as well as *V*_A_ as a high proportion of *V*_T_ during exercise is preferred for facilitating gas exchange compared with excessive *f*_B_ (assuming adequate pulmonary blood volume/distribution in both instances) (Hey et al. 1966; Dempsey et al. 1984; Aaron et al. 1992; Kinker et al. 1992), the disproportionately lower *V̇*_A_ relative to *V̇*_E_ driven by decreased *V*_A_ in the Arg16Arg *β*_2_AR genotype indeed suggests these individuals demonstrated a smaller total alveolar surface area available for O_2_ transport compared with Arg16Gly and Gly16Gly variants.

In addition to potential effects of altered alveolar respiration on gas exchange and O_2_ transport in adults demonstrating the Arg16Arg *β*_2_AR genotype, we acknowledge that metabolic pathways involving changes to processes of both glycolysis and lipolysis have been separately linked to SNPs of the ADRB2 (Kjaer et al. 1985; Wahrenberg et al. 1987; Large et al. 1997). While not tested in this study, others suggest substitution of Arg for Gly at codon 16 of the ADRB2 (i.e., Gly16Gly or Arg16Gly) leads to increased *β*_2_AR agonist affinity associated with adipocytes (Large et al. 1997). Thus, in theory, it is possible the Arg16Arg *β*_2_AR genotype in this study indeed contributed to low receptor sensitivity to the sympathomimetic effects of exercise. Following could have been lesser than expected lipolytic function in Arg16Arg variants, and thereby a muted ability to preserve glucose for oxidation culminating in reduced peak workload and economy of substrate oxidation compared with Arg16Gly and Gly16Gly *β*_2_AR genotypes (Kjaer et al. 1985; Wahrenberg et al. 1987; Large et al. 1997).

Our initial observations indeed suggest worse aerobic capacity (i.e., both workload and *V̇*O_2_) in Arg16Arg compared with Arg16Gly and Gly16Gly *β*_2_AR genotypes, which could have been used to readily explain group differences for *V̇*_A_ and *V̇*O_2_ during subsequent exercise testing at low and modest relative workload intensities. Nevertheless, we highlight that along with decreased workload and *V̇*O_2_ during submaximal exercise, compared with Arg16Gly and Gly16Gly *β*_2_AR genotypes, Arg16Arg variants also demonstrated reduced *V*_A_, *V̇*_A_/*V̇*_E_, and *V*_A_/FVC coupled with increased *V*_D_/*V*_T_. These collective respiratory responses in Arg16Arg variants do not resemble changes consistent with individuals performing the lowest workloads at modest or moderate intensity exercise in this study. Therefore, we suggest low external workload and potential effects of SNPs of the ADRB2 on metabolic pathways cannot by themselves explain unique responses of *V̇*O_2_, EC, EC_NET_, and alveolar respiration (i.e., *V*_A_, *V̇*_A_/*V̇*_E_, etc.) in adults demonstrating the Arg16Arg *β*_2_AR genotype.

#### Limitations

In addition to not directly assessing *β*_2_AR expression and function or measuring lung fluid changes during exercise, we acknowledge that we are unable to directly account for intramuscular factors related to microvasculature (e.g., convection, conduction, etc.) and bioenergetics (e.g., mitochondrial function/density, oxidative enzymes, etc.,) in the interpretation of our gross substrate oxidation data. Use of invasive (e.g., skeletal muscle biopsy) and non‐invasive (e.g., near‐infrared spectroscopy) methods in future work may help to refine the understanding of the intersecting contributions of skeletal muscle bioenergetic adaptations involved in the O_2_ transport chain influential to oxidative capacity as these factors relate with SNPs of the ADRB2. We also recognize that in addition to SNPs at codon 16 of the ADRB2 there are other SNPs at different codons that have been genotyped (e.g., position 27) (Large et al. 1997; Dishy et al. 2001), which may be influential as complex haplotype effects for the hypothesis tested in this study. Nevertheless, compared with the strength of proposed effects of SNPs at codon 16 of the ADRB2 on cardiopulmonary responses to exercise, based on evidence to date, we suggest potential independent influences of SNPs at codon 27 of the ADRB2 would not be expected to explain these data (Large et al. 1997; Dishy et al. 2001; Garovic et al. 2003; Snyder et al. 2006a,b). Our sample sizes respective of each SNP at codon 16 of the ADRB2 were powered to detect physiological differences associated with variability for this single allele (Snyder et al. 2006a). Lastly, we acknowledge that for there to be any possibility for the clinical translation of these data (e.g., heart failure, asthma, etc. (Spina et al. 1989; Van Iterson et al. 2015; Wagoner et al. 2000)), large scale follow‐up studies in humans must be performed that include comprehensive genotyping of all allele interactions associated with the ADRB2 as they relate with exercise phenotypes.

## Conclusions

These data suggest for the first time that for a given submaximal exercise *V̇*_E_, healthy adults expressing the Arg16Arg *β*_2_AR genotype demonstrate blunted elevations in V_A_ and V̇_A_ coupled with reduced economy of substrate oxidation compared with Arg16Gly and Gly16Gly variants. Accordingly, because in the lung there is a predominating density and distribution of *β*_2_ARs on alveolar tissue and there is a specific role these receptors play in helping to maintain alveolar surface area needed for proper gas exchange (Carstairs et al. 1985; McGraw et al. 2001; Sartori et al. 2002; Mutlu et al. 2004; Snyder et al. 2006d, 2007), these are hypothesis generating data suggesting the Arg16Arg SNP of the ADRB2 may be associated with decreased total alveolar surface area available for gas exchange during submaximal exercise in some, but not all healthy adults. Upon confirmation of mechanisms proposed in this study following completion of more advanced genomic and exercise phenotype studies in future work, there are potential clinical implications tied to the putative link between SNPs of the ADRB2 and oxidative capacity associated with alveolar respiration in patients with cardiopulmonary diseases (e.g., asthma, heart failure, etc. (Snyder et al. 2006d; Spina et al. 1989; Van Iterson et al. 2015; Wagoner et al. 2000)) for whom pharmacotherapies including *β*_2_AR agonists or blockers are considered part of the routine standard of care.

## Conflict of Interest

The authors of this manuscript have no conflicts of interest to disclose.

## Appendix 1

### Calculation methods involving use of “ideal” alveolar air equations

By using arterial gas measurements and acquired breath‐by‐breath respiratory gas exchange and volume flow responses, “ideal” alveolar air equations and associated parameters accounting for body temperature [for PaCO_2_; PaCO_2_ × (10^0.021×(*T*‐37)^ (Siggaard‐Andersen 1974); and for PaO_2_ using equations of Severinghaus (Severinghaus 1979)] could be used to calculate the following variables (as discussed above in methods) (Riley and Cournand 1951; Severinghaus 1966; Van Iterson et al. 2017b):alveolar ventilation,

$${\dot{V}}_{A}\left(\text{BTPS}\right)=\frac{\left[0.760\times \left(273+T\right)÷273\right]\times \dot{V}O_{2}\left(\text{STPD}\right)}{{\text{PIO}}_{2}-{\text{PaO}}_{2}}$$

alveolar volume,

$$V_{A}={\dot{V}}_{A}\left(\text{BTPS}\right)÷f_{B}$$

alveolar O_2_ tension,

$${\text{PAO}}_{2}={\text{PIO}}_{2}+\frac{{\text{PACO}}_{2}\times {\text{FIO}}_{2}\times \left(1-\text{RER}\right)}{100\times \text{RER}}-\frac{{\text{PACO}}_{2}}{\text{RER}}$$

alveolar CO_2_ tension,

$${\text{PACO}}_{2}=\frac{\left[0.760\times \left(273+T\right)÷273\right]\times \dot{V}{\text{CO}}_{2}\left(\text{STPD}\right)}{{\dot{V}}_{A}\left(\text{BTPS}\right)}$$

respiratory exchange ratio,

$$\text{RER}=\frac{{\text{PACO}}_{2}\times \left(1-{\text{FIO}}_{2}\right)}{{\text{PIO}}_{2}-{\text{PAO}}_{2}-{\text{FIO}}_{2}\times {\text{PACO}}_{2}}$$

inspired O_2_ tension,

$${\text{PIO}}_{2}={\text{FIO}}_{2}\times \left(P_{B}-47\right)$$

physiological dead space to tidal volume ratio,

$$\frac{V_{D}}{V_{T}}=\left(1-\frac{\left[0.760\times \left(273+T\right)÷273\right]\times \dot{V}{\text{CO}}_{2}\left(\text{STPD}\right)}{{\dot{V}}_{E}\left(\text{BTPS}\right)\times {\text{PACO}}_{2}}\right)$$

For above parameters related to “ideal” alveolar air equations: *V̇*_A_ is alveolar ventilation; *V*_A_ is alveolar volume; *f*_B_ is breathing frequency; *V*_D_ is physiological dead space, *V*_T_ is tidal volume; *P*_B_ is barometric pressure; 47 is lung water vapor pressure; *T* is body temperature in °C; RER is respiratory exchange ratio at the lung; PaCO_2_ is arterial CO_2_ tension; PACO_2_ is alveolar CO_2_ tension; PAO_2_ is alveolar O_2_ tension; FIO_2_ is inspired fraction of O_2_ equal to room air at sea level; PIO_2_ is inspired O_2_ tension; [0.760 × (273 + *T*)÷273] is the constant needed when computing partial pressure from fractional concentration involving both volumes/gas (STPD, standard temperature and pressure dry) and volumes/flows (BTPS, body temperature and pressure saturated) standards of measurement.
